# The extracellular matrix protein fibulin-3/EFEMP1 promotes pleural mesothelioma growth by activation of PI3K/Akt signaling

**DOI:** 10.3389/fonc.2022.1014749

**Published:** 2022-10-11

**Authors:** Arivazhagan Roshini, Chandra Goparaju, Somanath Kundu, Mohan S. Nandhu, Sharon L. Longo, John A. Longo, Joan Chou, Frank A. Middleton, Harvey I. Pass, Mariano S. Viapiano

**Affiliations:** ^1^ Department of Neuroscience and Physiology, State University of New York - Upstate Medical University, Syracuse, NY, United States; ^2^ Department of Cardiothoracic Surgery, Langone Medical Center, New York University School of Medicine, New York, NY, United States; ^3^ Department of Neurosurgery, State University of New York - Upstate Medical University, Syracuse, NY, United States

**Keywords:** mesothelioma, extracellular matrix, tumor microenvironment, PI3k signaling, antibody therapy, fibulin family

## Abstract

Malignant pleural mesothelioma (MPM) is an aggressive tumor with poor prognosis and limited therapeutic options. The extracellular matrix protein fibulin-3/EFEMP1 accumulates in the pleural effusions of MPM patients and has been proposed as a prognostic biomarker of these tumors. However, it is entirely unknown whether fibulin-3 plays a functional role on MPM growth and progression. Here, we demonstrate that fibulin-3 is upregulated in MPM tissue, promotes the malignant behavior of MPM cells, and can be targeted to reduce tumor progression. Overexpression of fibulin-3 increased the viability, clonogenic capacity and invasion of mesothelial cells, whereas fibulin-3 knockdown decreased these phenotypic traits as well as chemoresistance in MPM cells. At the molecular level, fibulin-3 activated PI3K/Akt signaling and increased the expression of a PI3K-dependent gene signature associated with cell adhesion, motility, and invasion. These pro-tumoral effects of fibulin-3 on MPM cells were disrupted by PI3K inhibition as well as by a novel, function-blocking, anti-fibulin-3 chimeric antibody. Anti-fibulin-3 antibody therapy tested in two orthotopic models of MPM inhibited fibulin-3 signaling, resulting in decreased tumor cell proliferation, reduced tumor growth, and extended animal survival. Taken together, these results demonstrate for the first time that fibulin-3 is not only a prognostic factor of MPM but also a relevant molecular target in these tumors. Further development of anti-fibulin-3 approaches are proposed to increase early detection and therapeutic impact against MPM.

## Introduction

Malignant pleural mesothelioma (MPM) is a rare and aggressive type of cancer, with a worldwide incidence ranging from 10 to 20 new cases per million people per year (1). MPM is largely caused by exposure to asbestos, which is still used in a variety of commercial products. In addition, new fibrous nanomaterials, such as carbon nanotubes and other nano-particulates, have been recently highlighted as potential etiological agents that could contribute to the future incidence of this cancer (2). The extended tumor latency, difficulty in early diagnosis, and limited therapeutic choices contribute to the high lethality of MPM, which has a median survival of 12 months (3, 4). Improvements in early detection and targeted therapies are direly needed to increase the efficacy of treatments given to patients with these tumors.

Several circulating proteins and RNA molecules have been proposed as diagnostic and prognostic biomarkers of MPM (5–9) but only the glycoprotein mesothelin has been approved by the US Food and Drug Administration as a biomarker used to determine tumor response to treatment (10). However, measurement of mesothelin alone is considered insufficient as a sensitive indicator of tumor progression, which has prompted the search for additional molecules that could be combined to establish a biomarker signature with prognostic value.

Fibulin-3 (gene *EFEMP1*) is an extracellular matrix (ECM) protein involved in organizing the ECM scaffold and promoting cell-ECM adhesion in normal connective tissues (11, 12). Fibulin-3 is elevated in pleural effusions from MPM patients and has been used to distinguish these patients from normal individuals and from those who have non-malignant pleural inflammation (13, 14). Comparative analysis of fibulin-3 and mesothelin has suggested that mesothelin correlates with MPM diagnosis more accurately than fibulin-3 but the latter could be a better prognostic factor (15, 16).

Fibulin-3 is upregulated in several types of solid cancers, including genito-urinary, colorectal, pancreatic, bone, and primary brain tumors (gliomas) (17–23). This upregulation has been correlated with accelerated tumor growth and invasion, suggesting a tumor-promoting function for this ECM protein (22, 24–26). However, some studies have described tumor-suppressing effects of fibulin-3, most notably in lung carcinoma (27), thus underscoring the importance of studying both the expression and the functions of this protein in mesothelial tumors surrounding the lungs. Circulating fibulin-3 has been proposed as a biomarker candidate for MPM, both by primary studies (13, 28–30) and meta-analyses (31–33) of patient populations; however, the functions and potential mechanisms of fibulin-3 in this cancer type remain completely unknown.

Here, we report for the first time the tumor-promoting effects of fibulin-3 in MPM and the molecular mechanisms and genetic programs activated by this protein in MPM cells. In addition, we demonstrate the anti-tumor efficacy of downregulating fibulin-3 mRNA and targeting fibulin-3 protein with a novel function-blocking antibody. These studies extend the utility of fibulin-3 as a potential biomarker for MPM and demonstrate that this protein is, more importantly, a relevant molecular target in these tumors.

## Materials and methods

### Cells and tissue specimens

MPM tumors and normal mesothelial tissues were procured from the Early Detection Research Network - Mesothelioma Biomarker Discovery Laboratory (New York University Langone Medical Center), after approval by the institutional review board and recording of patient consent for research. The non-malignant human mesothelial cell line LP-9 was obtained from Coriell Institute for Medical Research (Camden, NJ) and the cell lines H226 and MSTO211H were from American Type Culture Collection (ATCC, Manassas, VA). The cell lines H2595, H2596, H2452, H2373, H2461, HP1 and HP3 were originally generated at New York University (H.P.) and have been previously described (34, 35). Short-tandem repeat identification of the cells was performed at the University of Arizona Genetics Core and is provided in **Supplementary Table I**. All the cell lines were regularly cultured in DMEM medium containing 4.5 g/L glucose, 10% v/v fetal bovine serum, and standard penicillin and streptomycin antibiotics.

### DNA, antibodies, and biochemical reagents

The full-length clone of human fibulin-3 (1,479 bp, 453 amino acids) and siRNAs/shRNAs against human fibulin-3 have been previously characterized (17, 36). Two independent RNAi sequences were validated in MPM cells and confirmed to downregulate fibulin-3 expression and fibulin-3-dependent signaling (**Supplementary Figure 1**); further results in this study are representative of single RNAi sequences for improved clarity. The sequences of RNAi oligonucleotides and primers for semiquantitative RT-PCR (qRT-PCR) are listed in **Supplementary Table II**. Lentiviral particles carrying firefly Luciferase *fLuc* (Genecopoeia LPP-hLUC-LV206; Rockville MD) were used to generate stably transduced cells for *in vivo* studies.

The function-blocking monoclonal antibody *mAb428.2*, developed against an N-terminal motif of human fibulin-3, has been previously validated for anti-tumor effects in fibulin-3-expressing solid tumors (37). This antibody was sequenced and the V_H_ and V_L_ sequences were cloned into a human IgG1 backbone, resulting in a new chimera antibody for the present study. Chimera mAb428.2 was routinely produced and purified in low-endotoxin conditions (less than 0.5 EU/mg) by Absolute Antibody Ltd (Oxford, UK). The antibody was confirmed to have the same or better ability to detect and block fibulin-3 as the original mouse antibody. Low-endotoxin, control human IgG was purchased from Molecular Innovations (Novi, MI) and prepared in the same conditions as mAb428.2. All other antibodies used in this study are listed in **Supplementary Table III**. The potent PI3K inhibitor LY294002 (Tocris, Minneapolis MN) was dissolved in dimethyl sulfoxide and used at a final concentration of 20 µM in MPM cultures as described (38).

### Biochemical assays and immunohistochemistry

Fibulin-3 expression in MPM cells was quantified by qRT-PCR and confirmed by indirect ELISA using a commercially available kit (USCN Life Science, Wuhan, China) as previously described (13). Fibulin-3 was detected in tissue specimens by reverse-transcription PCR and immunohistochemistry following previously detailed protocols (39). Cell proliferation in tissues was determined by immunostaining for the nuclear antigen Ki67 (17). Ki67-positive nuclei were quantified in tumor sections of similar size and cell density using the software ImageJ. Transfection of cells with cDNA or siRNAs was performed using Lipofectamine 2000 or RNAiMAX reagents (Invitrogen), respectively, following the manufacturer’s instructions. Culture media was routinely changed to serum-free Opti-MEM medium the day after transfection, followed by cell collection after 24 hours. Samples were subsequently processed for qRT-PCR or Western blotting following standard procedures. All transfections were performed at a cell density of 1 x 10^6^ cells/ml.

### Dataset analysis

To perform whole transcriptome profiling, cells were transfected with anti-fibulin-3 or control siRNAs (three independent replicates for each transfection) and incubated for 48 hours as described above. Total RNA was extracted from frozen cell pellets using Pure Link RNA mini kit (Invitrogen) and enriched for poly-A RNA using Ribo-Zero Globin kit (Illumina, San Diego, CA). Libraries were prepared using the TruSeq Stranded RNA kit v2 (Illumina) and sequenced in an Illumina NextSeq 500 instrument with 1 x 75 bp single-end reads and a targeted averaged depth of coverage >25 million reads per sample. Sequencing reads were aligned to the human genome (hg38) and quantified using the Partek E/M tool provided by Partek Flow software (Partek, St Louis, MO). Gene counts were processed to remove cell line-dependent effects (batch-effects) and subsequently compared by analysis of co-variance to examine the main effect of treatment (siRNA). Differences in gene expression between control and fibulin-3 knockdown were considered significant at a false discovery rate-correct value q < 0.05. Genes showing a difference >1.5-fold between treatments were analyzed using the Kyoto Encyclopedia of Genes and Genomes (KEGG) and Ingenuity Pathway Analysis software (Qiagen) to identify functional pathway enrichment. FASTQ RNAseq files and processed gene and transcript counts have been deposited in the NCBI Gene Expression Omnibus (GSE133933). Normalized gene expression datasets from different cancer types (**Supplementary Table IV**) were obtained from The Cancer Genome Atlas (*TCGA*) through the Broad Institute Firehose repository. Correlation of fibulin-3 expression against tumor grade, stage, metastatic features, and histology was performed using the TCGA mesothelioma dataset (N=87) that has been recently characterized (40).

### *In vitro* assays

Cell behavior was studied in MPM cell lines with high fibulin-3 expression (H2595, HP1, or H226), low fibulin-3 expression (H2596), and normal mesothelial cells (LP-9). To quantify cell viability, cells were seeded at a density of 4x10^4^ cells/well in 96-well plates and analyzed after 48 (H2595, HP1) or 72 hours (LP-9, H2596) using a luminescent assay to measure ATP production (Cell Titer-Glo kit Promega). For colony formation in soft-agar, 5 x 10^3^ cells were mixed with a solution of 1% agarose in culture medium and seeded in 35 mm dishes. After two weeks, the colonies were stained with crystal violet and quantified (41). Two-dimensional cell migration assays were performed as conventional monolayer gap-closure assays in 6-well plates. A gap was produced in confluent cell monolayers using a sterile pipette tip and the gap closure was measured after 24 hours using brightfield microscopy. For cell invasion assays, 5 x 10^4^ cells were seeded on Matrigel™-coated Transwell™ inserts (8 µM pore diameter, BD Sciences) and allowed to invade the matrix for 48 hours. Transmigrated cells were fixed, stained with crystal violet, and counted under a microscope. Detailed methods for these assays have been previously described (41).

### *In vivo* studies

All animal experiments were performed using 8-week-old athymic nude mice (FoxN1^nu/nu^, Envigo), following approval by the institutional animal care and use committee at SUNY Upstate Medical University. MPM cells were orthotopically implanted in the pleural space following a previously described model (42). Briefly, animals were anesthetized with isoflurane and cells were injected using a 0.5 ml insulin syringe (28G needle) in the right antero-lateral pleural space between the fourth and fifth ribs, 3-4 mm to the right of the sternum and to a depth of 4 mm. Cells (2x10^6^ H226 or 4x10^6^ H2595, expressing fLuc) were resuspended in 100 µL PBS. Tumor growth was monitored by bioluminescence and antibody treatment started when tumors had an integrated radiance signal above 1x10^5^ photons/sec x cm^2^. Mice showing fLuc signal in the peritoneal cavity were considered mis-injected and discarded. mAb428.2 (30 mg/kg x day) or control human IgG were delivered by intrapleural injection for two weeks (5 days on + 2 days off). Tumors were monitored by bioluminescence for an additional month after treatment and mice were euthanized when they showed significant weight loss or additional symptoms of tumor burden. Due to the slow-growing nature of these tumors and difficulty assessing their thoracic spread, survival experiments were finished at 15 weeks (105 days).

### Statistics

All the *in vitro* experiments were repeated in triplicate with three independent replicates per condition. *In vivo* studies used N>=10/arm for survival studies and N=5/arm for fixed-endpoint histology. Histological and bioluminescence signals from *in vivo* studies were quantified blindly by separate investigators. Values in the graphs represent mean ± SD. Grouped results were analyzed by one- or two-way ANOVA depending on the experimental design. Experiments in which some replicates were unavailable (e.g., tumors that did not yield sufficient RNA) were analyzed by mixed-effects model with false-discovery correction (Benjamini-Hochberg). All differences were deemed significant at p<0.05.

## Results

### Fibulin-3 is upregulated in MPM cells and tissues

**Figure 1:**
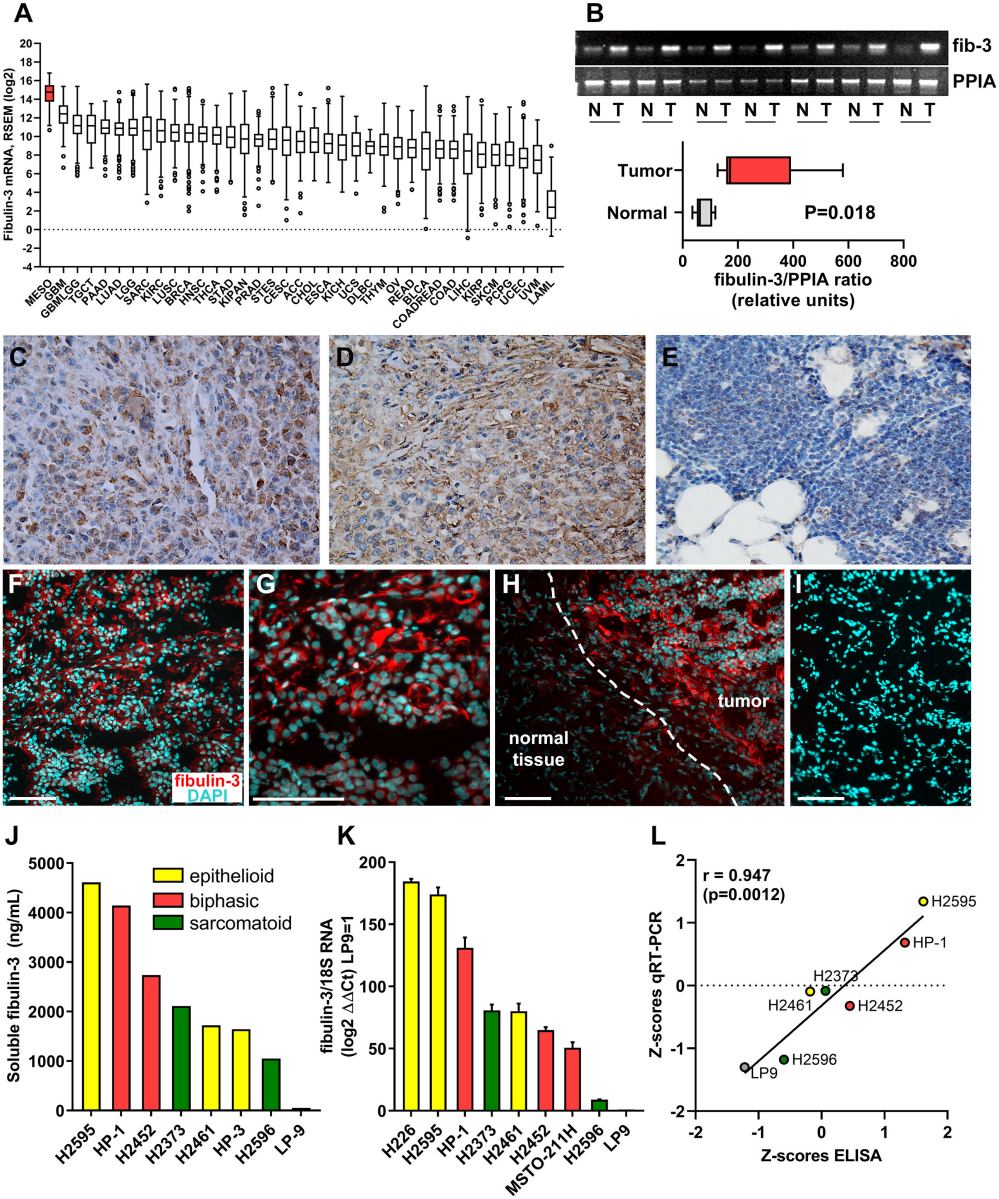
*Fibulin-3 is upregulated in MPM cells and tissues*. **(A)** Abundance of fibulin-3 (*EFEMP1*) mRNA in solid and liquid tumors available from TCGA (data queried from Broad GDAC Firehose); the full name and number of cases for each tumor type are provided in **Supplementary Table IV**. **(B)** Comparative mRNA expression of fibulin-3 (*fib-3*) and the loading control cyclophilin (*PPIA*) for MPM tumor (*T*) and normal adjacent-to-tumor (*N*) tissues. **(C-E)** Conventional immunohistochemical detection of fibulin-3 in epithelioid MPM **(C-D)** and normal pleura **(E)**; images captured at 400x magnification. **(F-I)** Immunofluorescence detection of fibulin-3 in epithelioid MPM **(F-G)** and adjacent normal tissue **(H)**; notice the abundant pericellular staining only in the tumor tissue; this staining is specific and not observed in absence of primary antibody **(I)**. Bars= 100 µm. **(J-L)** Fibulin-3 expression in MPM cells (originated from different tumor subtypes) and normal mesothelial cells was detected by ELISA for the protein secreted to the culture medium **(J)** or by qRT-PCR **(K)**. Both measures showed significant positive correlation **(L)**.

Circulating fibulin-3 has been detected in the blood and pleural effusions of MPM patients (13, 35) but this protein has not been quantified in the tumor mass and specifically in mesothelioma cells. In addition, there is no information about fibulin-3 expression in MPM, compared to other malignancies where the role of this ECM protein has been described. Therefore, we first compiled gene-expression data available through the TCGA Program to analyze the expression of fibulin-3 in samples from 36 types of solid and liquid tumors. This analysis revealed that malignant mesothelioma is the cancer type with highest absolute expression of fibulin-3 (**Figure 1A**), potentially explaining why this protein is readily found in liquid biopsies of MPM. We confirmed the upregulation of fibulin-3 mRNA in MPM specimens compared to normal pleura (**Figure 1B**) as well as the elevated expression of fibulin-3 protein in tumor tissues and its sharp decline in normal pleural tissue adjacent to tumor (**Figure 1C-I**). Analysis of available TCGA data suggested a higher expression of fibulin-3 in epithelioid mesothelioma compared to other histological subtypes (**Supplementary Figure 2**), matching our observation of intense fibulin-3 staining in epithelioid specimens (**Figures 1C, D**). However, TCGA data did not show a correlation of fibulin-3 expression with tumor stage or grade (**Supplementary Figure 2**) in agreement with prior observations suggesting that fibulin-3 expression is insufficient for differential diagnosis of MPM. Further analysis in cultured MPM cells confirmed that fibulin-3 is upregulated in mesothelioma cells compared to normal mesothelial cells (**Figures 1J-L**). As observed in tissues, MPM cells derived from epithelioid tumors tended to have the highest expression of fibulin-3, although there was significant variability of expression likely caused by the original expression in the tumor as well as clonal selection during culture. We found excellent correlation between mRNA and secreted protein expression, suggesting that either the mRNA or circulating protein levels could be used as comparable measures of fibulin-3 expression in the tumor.

### Fibulin-3 promotes MPM cell viability and invasion

**Figure 2:**
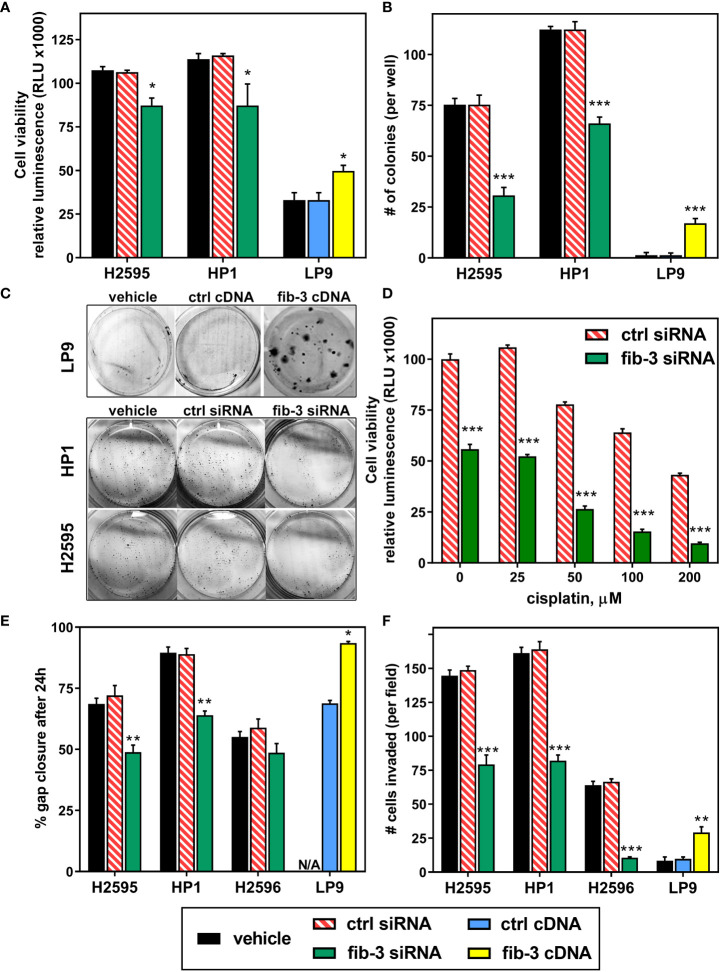
*Fibulin-3 promotes MPM cell viability and invasion*. **(A)** Fibulin-3 (*fib-3*) knockdown in MPM cells (H2595 and HP1) reduced cell viability, whereas fibulin-3 overexpression in normal mesothelial cells (LP-9) increased it. **(B, C)** The same effects were observed on the clonogenicity of MPM and mesothelial cells after knockdown or overexpression of fibulin-3, respectively. **(D)** Transient knockdown of fibulin-3 in H2595 cells significantly increased their sensitivity to cisplatin. **(E-F)** Fibulin-3 deficiency also reduced the two-dimensional migration **(E)** and three-dimensional invasion **(F)** of H2595 and HP1 cells, as well as invasion of H2596 cells. Overexpression of fibulin-3 in LP-9 cells achieved the opposite effects (*N/A,* not assayed). Results were analyzed by 1-way ANOVA for each cell line **(A, B, E, F)** or two-way ANOVA (cisplatin x transfection) for cisplatin resistance **(D)**. *p<0.05; **p<0.01; ***p<0.001. *ctrl,* control transfection.

Although fibulin-3 is consistently elevated in MPM, no studies have investigated if this is merely a passenger phenomenon or may actually contribute to tumor malignancy. To address this question, we overexpressed full-length fibulin-3 in normal mesothelial cells (LP-9) and transiently knocked it down in two MPM cell lines that have abundant fibulin-3 expression (HP1 and H2595, derived from biphasic and epithelioid tumors, respectively). Fibulin-3 knockdown reduced MPM cell viability (**Figure 2A**) and had a marked negative effect on colony formation (**Figures 2B, C**). The decreased viability was further potentiated when fibulin-3 knockdown was combined with treatment of the cells with the standard chemotherapeutic cisplatin (**Figure 2D**). Although the cisplatin concentrations used are higher than those applicable in a clinical setting, this result strongly suggests that fibulin-3 contributes to MPM cell viability and may contribute to resistance to apoptosis-inducing chemotherapeutic agents. In agreement, overexpression of fibulin-3 in normal LP-9 cells increased their viability and ability to form colonies (**Figures 2A–C**).

Fibulin-3 has been shown to increase cell adhesion and migration in several solid tumor models (17, 18, 25). Accordingly, fibulin-3 knockdown reduced both the migration and invasion of MPM cells with high fibulin-3 expression (HP1 and H2595, **Figures 2E, F**). In a cell line with low fibulin-3 expression (H2596, sarcomatoid) fibulin-3 knockdown did not visibly impact motility –perhaps due to the slow motile nature of those cells– but significantly reduced cell invasion, suggesting that even MPM cells with modest fibulin-3 expression are sensitive to targeting of this protein. On the other hand, fibulin-3 overexpression potentiated the migratory phenotype of normal mesothelial cells, increasing both cell motility and invasion. Overall, our results from cell viability and migration assays demonstrate for the first time that upregulation of fibulin-3 in MPM is associated with phenotypic changes that conduce to tumor progression.

### Fibulin-3 effects are mediated by PI3K/Akt signaling in MPM cells

**Figure 3:**
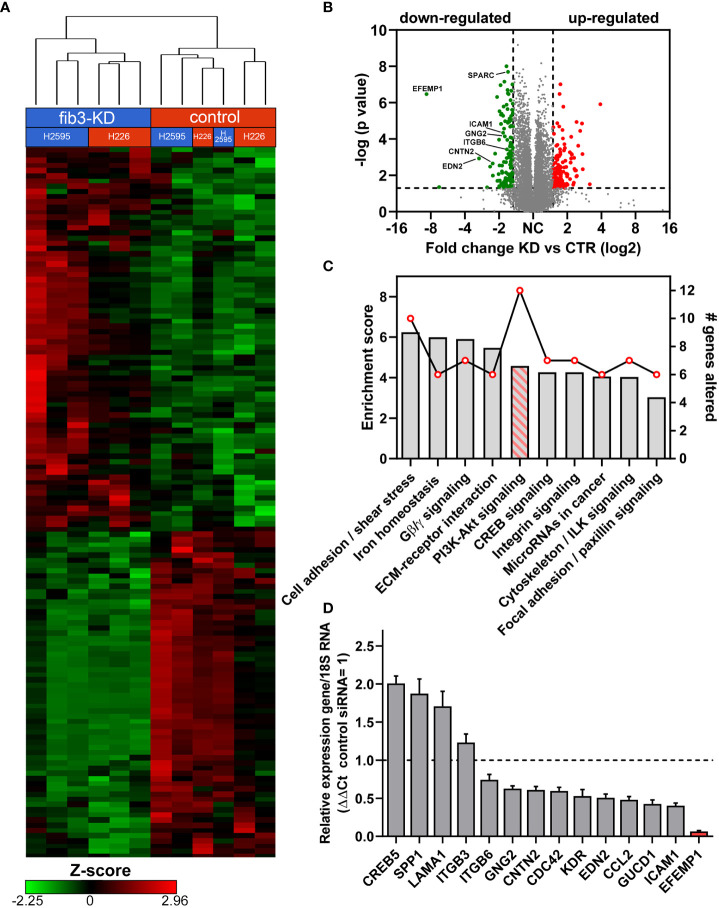
*Fibulin-3 expression correlates with an enhanced cell-adhesion and PI3K signaling signature.* **(A)** Gene expression clustering in two MPM cell lines after transient fibulin-3 knockdown (*fib3-KD*). Unsupervised cluster analysis revealed common genes downregulated in both cell lines. **(B)** Volcano plot of down- and up-regulated genes common to H2595 and H226 cells after fibulin-3 knockdown; several cell-adhesion molecules are indicated among the down-regulated genes. **(C)** Pathway-enrichment analysis revealed top enrichment scores (bars) for cell adhesion mechanisms. The line indicates the number of genes altered for each of those pathways after fibulin-3 knockdown, being highest for PI3K/Akt signaling. **(D)** Analysis of cell cell-adhesion genes in H2595 by qRT-PCR confirmed the downregulation of several of those genes after fibulin-3 knockdown (*EFEMP1* is the official symbol of fibulin-3 gene).

To identify potential molecular mechanisms regulated by fibulin-3 in MPM we knocked down this protein in two epithelioid MPM cell lines, H2595 and H226. Global transcriptome changes common to both cell lines were assessed by RNAseq followed by analysis of the most affected pathways (**Figures 3A–C**). Our results revealed a relatively small set of common genes affected by fibulin-3 knockdown in both cell lines (**Figure 3B**), several of which are involved in cell adhesion, ECM organization, and ECM-cell interaction (**Figure 3C**). Using qRT-PCR in parallel experiments we validated the downregulation of those genes (**Figure 3D** and **Supplementary Figure 3**), which included cell adhesion receptors (*CNTN2, ITGB6, ICAM1*) and transduction factors for cell adhesion signaling (*IQGAP1, CDC42, GNG2, GUCD1*).

**Figure 4:**
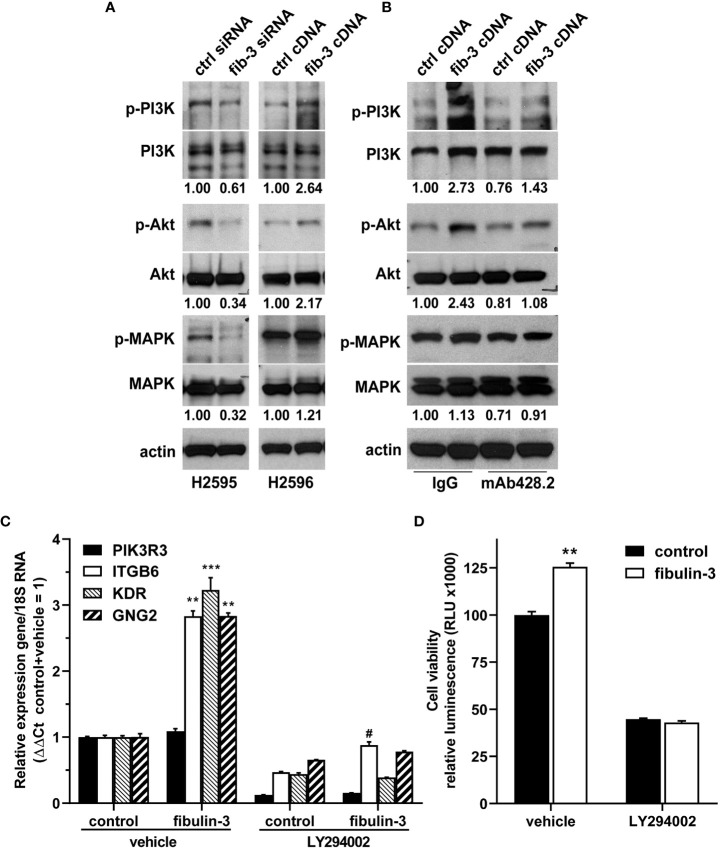
*Fibulin-3 regulates PI3K/Akt signaling in MPM cells.* **(A)** Transient knockdown of fibulin-3 (*fib-3*) in H2595 cells decreased the phosphorylation of PI3K regulatory subunit (p85), Akt, and p38 MAPK. Conversely, transient overexpression of fibulin-3 in H2596 cells increased the phosphorylation of PI3K and Akt (but had only minimal effect on MAPK). **(B)** The increased phosphorylation of PI3K/Akt by fibulin-3 in H2596 cells was blocked with anti-fibulin-3 mAb428.2 but not with a control antibody. The blots are representative images of experiments repeated in triplicate. The numbers under the blots represent the optical density ratio for the different phospho-bands referred to the control condition for each experiment (control = 1.00). Integrated optical density for each phosphoprotein was normalized to the total amount of that protein and to total actin used as loading control. Different profiles for total PI3K bands in panels **(A)** and **(B)** were the result of using different antibodies (**Suppl. Table III**). **(C)** Overexpression of fibulin-3 cDNA in H2596 cells increased the expression of PI3K-dependent genes (^**^p<0.01; ^***^p<0.001 significant differences in absence of LY294002, two-way ANOVA). This effect was prevented by addition of the PI3K inhibitor LY294002 (# p<0.05 significant differences in presence of LY294002, two-way ANOVA). **(D)** Overexpression of fibulin-3 increased the viability of H2596 cells at the 72-hour mark; this effect was also abolished by LY294002 (^**^p<0.01 by two-way ANOVA).

Pathway enrichment analysis suggested that several of these genes were involved in, or regulated by, PI3K/Akt signaling. In agreement with this analysis, knockdown of fibulin-3 in H2595 cells, which have high fibulin-3 expression, decreased the phosphorylation of PI3K (p85) and Akt, as well as MAPK (p38) (**Figure 4A**). Conversely, overexpression of fibulin-3 in the “low fibulin-3” cell line H2596 increased both phospho-PI3K and phospho-Akt, although not phospho-MAPK (**Figure 4B**). Importantly, activation of PI3K/Akt by fibulin-3 in MPM cells was significantly inhibited by our chimeric anti-fibulin-3 antibody, mAb428.2 (**Figure 4B**).

To further determine if PI3K signaling was underlying fibulin-3 effects in MPM cells, we repeated the overexpression of this protein in H2596 cells and confirmed that it upregulated PI3K-dependent genes (*ITGB6, KDR, GNG2*), an effect that was abolished by the PI3K inhibitor LY294002 (**Figure 4C**). Moreover, the PI3K inhibitor abolished the enhancing effect of fibulin-3 on H2596 cell viability in culture (**Figure 4D**). Taken together, these results indicate that fibulin-3 activates PI3K/Akt signaling in MPM cells, which is necessary for the pro-tumoral effects of this ECM protein.

### Targeting of fibulin-3 reduces MPM cell proliferation and tumor growth *in vivo*

**Figure 5:**
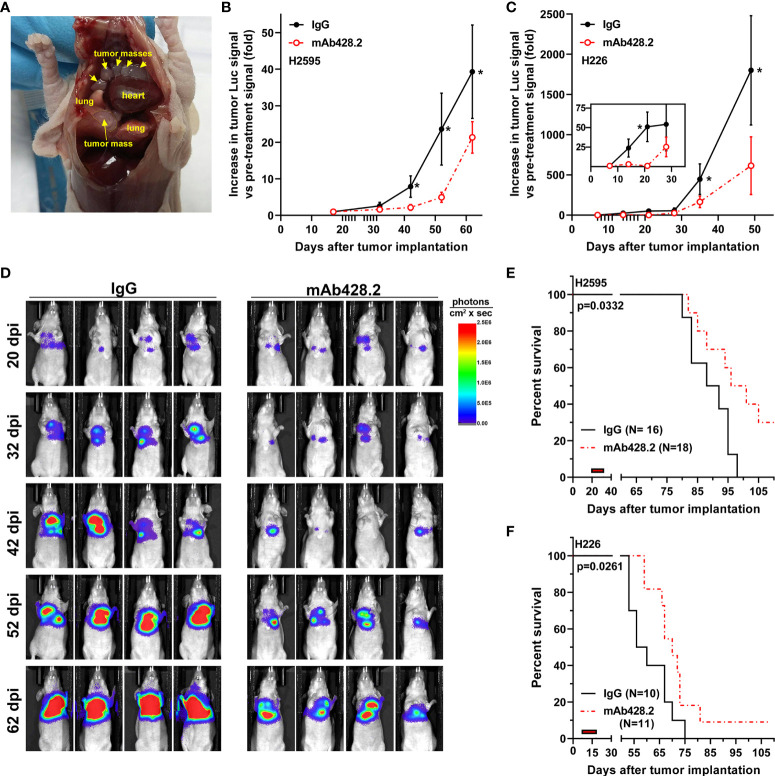
*Anti-fibulin-3 antibody reduces in vivo tumor growth and extends animal survival.* **(A)** Orthotopic MPM implantation revealed multiple tumor masses (arrows) attached to the inner lining of the pleura and surrounding the lungs. **(B-C)** Radiance measurements of fLuc-expressing H2595 **(B)** and H226 **(C)** intrapleural tumors. Because of differences in chest volume, tumor spread, and total radiance between animals, the radiance values (photons/sec x cm2) were calculated for each animal relative to the same animal before starting treatment (pre-treatment radiance = 1). Tick marks on the horizontal axis indicate treatment days post-implantation (20-31 dpi for H2595; 7-18 dpi for H226); the inset in **(C)** highlights the early response of H226 tumors to mAb428.2 during treatment. Results were analyzed by 1-way ANOVA for repeated measures (^*^p< 0.05). **(D)** Representative images of mice carrying H2595 tumors treated with mAb428.2 or control IgG. Mice were imaged on the first day of treatment (20 dpi) and for one month after being treated (32-62 dpi). **(E-F)** Treatment with mAb428.2 (two weeks at 30 mg/kg.day, red bars) extended the median survival of MPM-bearing mice (results analyzed by log-rank test). This anti-fibulin-3 treatment resulted in long-term survivors with reduced or stable tumor signal at the end of the 15-week experiment.

Encouraged by the results demonstrating that fibulin-3 has pro-tumoral functions on MPM cells, we tested whether this ECM protein would be a suitable therapeutic target *in vivo*. We used two MPM models with high fibulin-3 expression (fLuc-expressing H2595 and H226 cells), which were implanted in the pleural space. Tumors derived from these cells mimicked clinically relevant features, such as spreading throughout the chest cavity and growing slowly until they eventually wrapped around the lungs and pericardium (**Figure 5A**).

Once tumors were detectable by bioluminescence, they were treated by local injection of mAb428.2 daily for two weeks, using a dosage of 30 mg/kg that was previously shown to work against other fibulin-3-expressing solid tumors (37). The antibody exerted a strong cytostatic effect on the tumors, including significant reduction of tumor luminescence that extended for at least a month after the end of the injections (**Figures 5B–D**). This anti-tumor effect resulted in significant extension of animal survival in both MPM models (**Figures 5E, F**). In the case of H2595 tumors, 30% of the animals remained with reduced or stable disease by the end of the experiment (15 weeks post implantation). Orthotopic H226 tumors were more aggressive and resulted in shorter overall survival (**Figure 5F**); however, mAb428.2 treatment significantly impaired H226 tumor growth and resulted in a small batch of long-term surviving animals by the end of the experiment.

**Figure 6:**
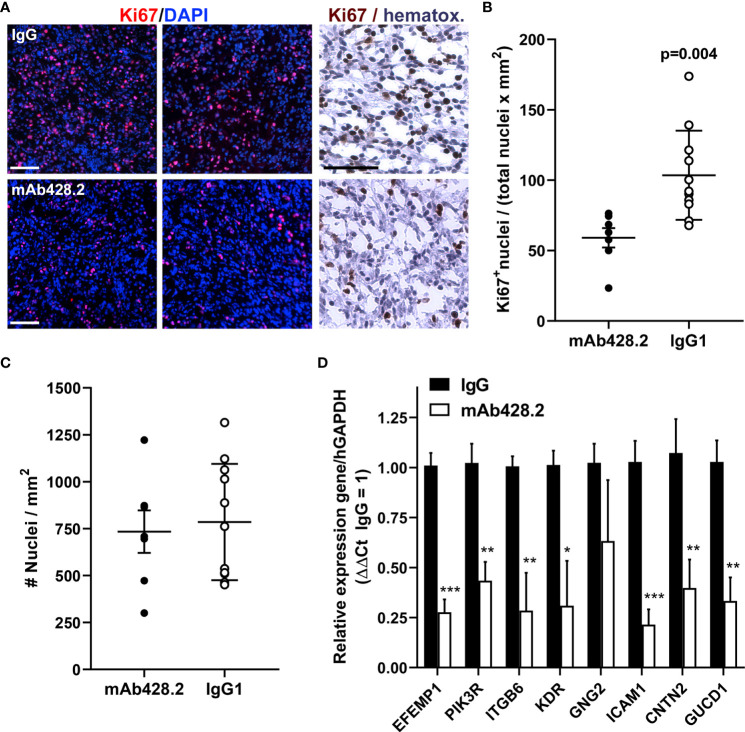
*Anti-fibulin-3 antibody reduces tumor cell proliferation in vivo.* **(A)** Representative images of intrapleural tumors (H226 cells) from mice euthanized one week after finishing antibody treatments. Ki67 expression is shown by immunofluorescence (counterstained with DAPI) and conventional DAB immunohistochemistry (counterstained with hematoxylin) at higher magnification. Notice the reduced Ki67 staining in mAb428.2-treated mice (bars= 100 µm). **(B)** Quantitative analysis of Ki67 immunofluorescent staining in intrapleural H226 tumors (N=5 mice/treatment, 3 sections/mouse); significant differences determined by Student’s t-test. **(C)** Internal control confirming that the tissues analyzed had comparable density of nuclei per area (average tumor area for imaging was 26.4 ± 8.5 mm2 and 28.4 ± 11.9 mm2 for IgG and mAb428.2 treatments, respectively). **(D)** Analysis of tumor tissue by qRT-PCR using human-specific primers; mAb428.2 treatment resulted in significant decrease in the expression of fibulin-3 (*EFEMP1*) and a subset of PI3K/Akt-regulated genes (^*^p<0.05; ^**^p<0.01; ^***^p< 0.001 as determined by mixed-effects analysis with false discovery rate (FDR>5%) correction).

To confirm the effects of mAb428.2, a number of mice carrying H226 tumors were euthanized at an early time point, one week after the last injection of antibody (25 days post implantation), and their tumors processed for immunohistochemistry or mRNA analysis. Animals treated with mAb428.2 showed significantly reduced intratumoral expression of the nuclear proliferation marker Ki67 (**Figures 6A–C**), confirming the anti-proliferative effect of mAb428.2 on MPM. In addition, qRT-PCR analysis confirmed that treatment with mAb428.2 had inhibited its target as expected. Tumors treated with this antibody showed significant reduction in the expression of fibulin-3 as well as a subset of PI3K/Akt-dependent genes (**Figure 6D**), matching our prior results in culture.

## Discussion

The low incidence and slow-forming nature of MPM are factors that keep these tumors undiagnosed until they are detected in an advanced stage that is resistant to therapy and has very poor survival rate (9). Conventional treatment continues to be limited to cytoreductive surgery followed by cisplatin/pemetrexed chemotherapy (43), nowadays combined with checkpoint-inhibitor immunotherapy (44). Nevertheless, conventional treatments still lack –or have minimal– supporting molecular information to identify responder patients or to direct the course of therapy (45). Molecular-targeted therapies for MPM have also been hampered by the limited availability of tumor samples and cell lines, as well as the slow growth and responses of MPM cells *in vitro* and *in vivo*, limiting the appeal of these cancer models for research. Furthermore, recent molecular studies have uncovered a considerable degree of inter- and intra-tumoral molecular heterogeneity in MPM (40, 46), revealing novel molecular subtypes and stem-like tumor cell populations that contribute to tumor growth and make MPM a moving target for molecular therapies. Accordingly, identification of novel molecular targets that can help overcome tumor heterogeneity and can be, at the same time, biomarkers of the disease will result in considerable improvements in patient selection, targeted therapy, and measurement of treatment response. This rationale has guided the development of targeted immunotherapies against mesothelin (47–49), a well-known biomarker expressed on the surface of mesothelioma cells. Mesothelin, however, has remained insufficient as a marker of tumor prognosis (15) and its functions in MPM and other solid tumors remain largely unknown.

Since the first description of fibulin-3 in pancreatic adenocarcinoma (24) and malignant glioma (17), this protein has been repeatedly demonstrated as a pro-tumoral factor in several cancer types, enhancing malignant features such as tumor cell proliferation, invasion and metastasis (17, 18, 20, 22, 25); tumor “stemness” (36, 50); angiogenesis (21, 39); and chemoresistance (36, 51). Several pathways have been postulated to underlie these functions of fibulin-3: A mechanism first observed in pancreatic cancer cells and later shown in other solid tumors involves activation of receptor tyrosine kinases (such as the EGF receptor), resulting in MAPK and Akt signaling (19, 24). In contrast, studies in highly invasive tumors such as osteosarcoma and malignant glioma have not reported activation of EGFR/MAPK by fibulin-3 but have shown instead direct activation of canonical TNF-alpha/NF-κB signaling (26, 36, 39). Strikingly, the functions or molecular mechanisms of fibulin-3 in MPM, the only cancer type where this protein has been validated as a tumor biomarker, have remained entirely unknown.

MPM is the only cancer type where secreted fibulin-3 has been detected in the blood and demonstrated to be specifically upregulated in patients with this malignant disease compared to normal individuals or patients with non-malignant pleural inflammation (13, 16, 52). The elevation of circulating fibulin-3 is likely a product of increased synthesis and secretion by the tumor cells rather than other cells in normal mesothelium, as suggested by our biochemical and immunohistochemical results (**Figure 1**). In agreement, Fibulin-3 has been shown to increase in normal mesothelial cells only after exposure to fluoro-edenite (53), a mineral fiber that facilitates the malignant transformation of these cells (54). Several studies have compared the expression of fibulin-3 against other candidate biomarkers in MPM, concluding that the detection of this protein alone may be insufficient to predict patient survival but can provide positive identification of MPM patients and could be useful for patient selection and stratification before treatment (55–57). It should be noted that fibulin-3 remains controversial as a biomarker (14), even though the original observations of circulating fibulin-3 in MPM patients in the US (13) have been validated in blinded fashion in population cohorts from Turkey (28), China (29) and Egypt (30). Accordingly, several meta-analyses have pointed out the value of fibulin-3 for positive identification of mesothelioma (31–33). Identifying the functions of fibulin-3 in MPM is therefore critical to determine whether upregulation of this protein truly contributes to tumor malignancy or is an epiphenomenon associated with tumor growth.

In this study, we have shown for the first time that fibulin-3 directly promotes the malignant behavior of MPM and can be targeted to reduce tumor growth. Fibulin-3 was highly expressed in MPM cells and tissues, although, as expected, data from the TCGA cohort did not reveal a particular correlation with tumor stage or metastasis. This could be the result of dysregulation of this gene shortly after malignant transformation (53), which would keep the expression of fibulin-3 elevated and contributing to tumor progression since the early stages of MPM growth (16). Indeed, our experimental results confirm that forced increase of fibulin-3 is sufficient to induce malignant traits in mesothelial cells, such as increased proliferation, clonogenicity, and invasion abilities, whereas downregulation of fibulin-3 in MPM cells decreases the same phenotypic traits.

At the molecular level, knockdown of fibulin-3 in MPM cells disrupted pathways involved in cell adhesion and cell-ECM interaction, matching the observed decrease in cell proliferation and migration/invasion. Pathway analysis also revealed that several of the cell-adhesion and motility genes downregulated in fibulin-3-deficient cells were under control of PI3K/Akt signaling. In agreement, we confirmed that fibulin-3 knockdown reduced PI3K/Akt as well as MAPK activation, whereas fibulin-3 overexpression increased both PI3K and Akt activation. This suggests that fibulin-3 regulates a PI3K/Akt/MAPK cascade that triggers MPM cell proliferation and motility, consistent with the role of this protein as an organizer of the ECM scaffold surrounding tumor cells (36, 58). More importantly, PI3K activation by fibulin-3 was entirely necessary for the effects of this ECM protein because both the enhancing effect of fibulin-3 on PI3K-dependent gene expression and MPM cell viability were abolished with a PI3K inhibitor. Taken together, these results indicate that fibulin-3 is sufficient to activate PI3K/Akt signaling in MPM cells and depends on this mechanism to exert its pro-tumoral effects in MPM. Although the overall mechanism of fibulin-3 in MPM is yet to be fully elucidated –in particular the identity of fibulin-3 receptors in MPM cells– these encouraging results establish a functional link between fibulin-3 upregulation in MPM and tumor malignancy, increasing the relevance of this protein from tumor biomarker to potential tumor target.

To further validate fibulin-3 as a clinically relevant target in MPM we performed animal studies targeting fibulin-3 with a function-blocking antibody that inhibits the signaling of fibulin-3 in MPM cells. This antibody is the mouse/human chimera version of a mouse monoclonal antibody (mAb428.2) designed to block an N-terminal motif of fibulin-3 that activates Notch/NF-κB signaling in malignant glioma cells (37). The fact that mAb428.2 blocks a different signaling mechanism in MPM cells suggests that the same motif of fibulin-3 may interact with different receptors in different tumor cells, leading to differential pathway activation. Alternatively, fibulin-3 may yet activate additional pathways in mesothelioma cells, not revealed in our study. In either case, our results indicate that blockade of the N-terminal domain of fibulin-3 is critical to inhibit its pro-tumoral functions. This domain is not involved in cell-ECM association but instead regulates the activity of metalloproteases such as ADAM17 (26, 37) and metalloprotease inhibitors such as TIMP-3 (59), both of which exert extensive control over ECM protein cleavage, activation of cell-membrane receptors, and release of soluble signals to the tumor microenvironment. Treatment of orthotopic MPM tumors with the chimera mAb428.2 inhibited malignant cell proliferation and reduced tumor growth, reflecting the inhibition of these potential signaling cascades. Importantly, tumor progression was stopped during treatment with mAb428.2 and only resumed well after the end of this treatment, increasing both the median survival and the number of long-term surviving animals. This suggests that fibulin-3 inhibition in MPM cells, even in cell types that secrete large amounts of this protein, has a sustained tumor-suppressive effect only limited by the availability of antibody and duration of our treatment.

Together with our *in vitro* results, our animal studies confirm that fibulin-3 not only promotes MPM progression but also can be targeted to prevent tumor growth. The unique location of this protein in the extracellular matrix opens new avenues for targeting the tumor microenvironment that is in intimate contact with tumor cells of different molecular makeup, which could help overcome the challenges of tumor cell heterogeneity and resistance. Antibodies against ECM proteins such as fibulin-3 would have the advantage of excellent accessibility to targets that are outside the tumor cells, as well as the possibility of being delivered locally in the pleural cavity, maximizing intratumoral accumulation. It is worth noting that recombinant antibodies against mesothelin, which has a cleaved soluble isoform that accumulates in the tumor matrix, have already been tested in the clinical setting (60). Similarly, chimeric antibodies against podoplanin, an ECM-associated protein that is another proposed MPM biomarker, have been validated in clinically-relevant MPM models (e.g., (61)).

In sum, our study shows for the first time the tumor-promoting functions, underlying mechanisms, and targeting relevance of fibulin-3 in MPM. Our results indicate that the upregulation of fibulin-3 is not a passenger event but a direct contributor to the malignant behavior of these tumors and therefore a target with translational potential. Future strategies could take advantage of the dual utility of this protein as a target and biomarker for MPM; patients could be stratified for treatment based on their circulating fibulin-3 levels, followed by treatment with anti-fibulin-3 agents. Anti-fibulin-3 targeted therapies in MPM may increase the efficacy of current treatments against these malignant tumors.

## Data availability statement

The datasets presented in this study can be found in online repositories. The names of the repository/repositories and accession number(s) can be found below: https://www.ncbi.nlm.nih.gov/geo/, GSE133933.

## Ethics statement

The animal study was reviewed and approved by SUNY Upstate Medical University institutional animal care and use committee (IACUC).

## Author Contributions

AR, CG, HP and MV conceived and designed this study. AR, CG, SK, MN, SL and JL developed methods, performed experiments, and acquired data. AR, CG, HP and MV analyzed and interpreted results. AR, CG, HP and MV wrote and revised the manuscript. HP and MV procured funding and supervised the project. All authors contributed to the article and approved the submitted version.

## Funding

This work was supported by research grants from the Department of Defense (CA-160356 and CA-170319) to MV and HP, and from the National Institutes of Health (NCI UO1-CA214195) to HP.

## Conflict of interest

MN and MV are co-inventors in the patent “Anti-fibulin antibodies and uses thereof” (USPTO 11,117,977/2021).

The remaining authors declare that the research was conducted in absence of any additional commercial or financial relationships that could be construed as potential conflict of interest.

## Publisher’s note

All claims expressed in this article are solely those of the authors and do not necessarily represent those of their affiliated organizations, or those of the publisher, the editors and the reviewers. Any product that may be evaluated in this article, or claim that may be made by its manufacturer, is not guaranteed or endorsed by the publisher.
