# Supplementary material for: The extracellular matrix protein fibulin-3/EFEMP1 promotes pleural mesothelioma growth by activation of PI3K/Akt signaling
Source: Front Oncol. 2022 Oct 11;12:1014749. doi: 10.3389/fonc.2022.1014749 (PMC9593058; doi:10.3389/fonc.2022.1014749)
Supplement: Supplementary file 1 [file DataSheet_1.pdf]

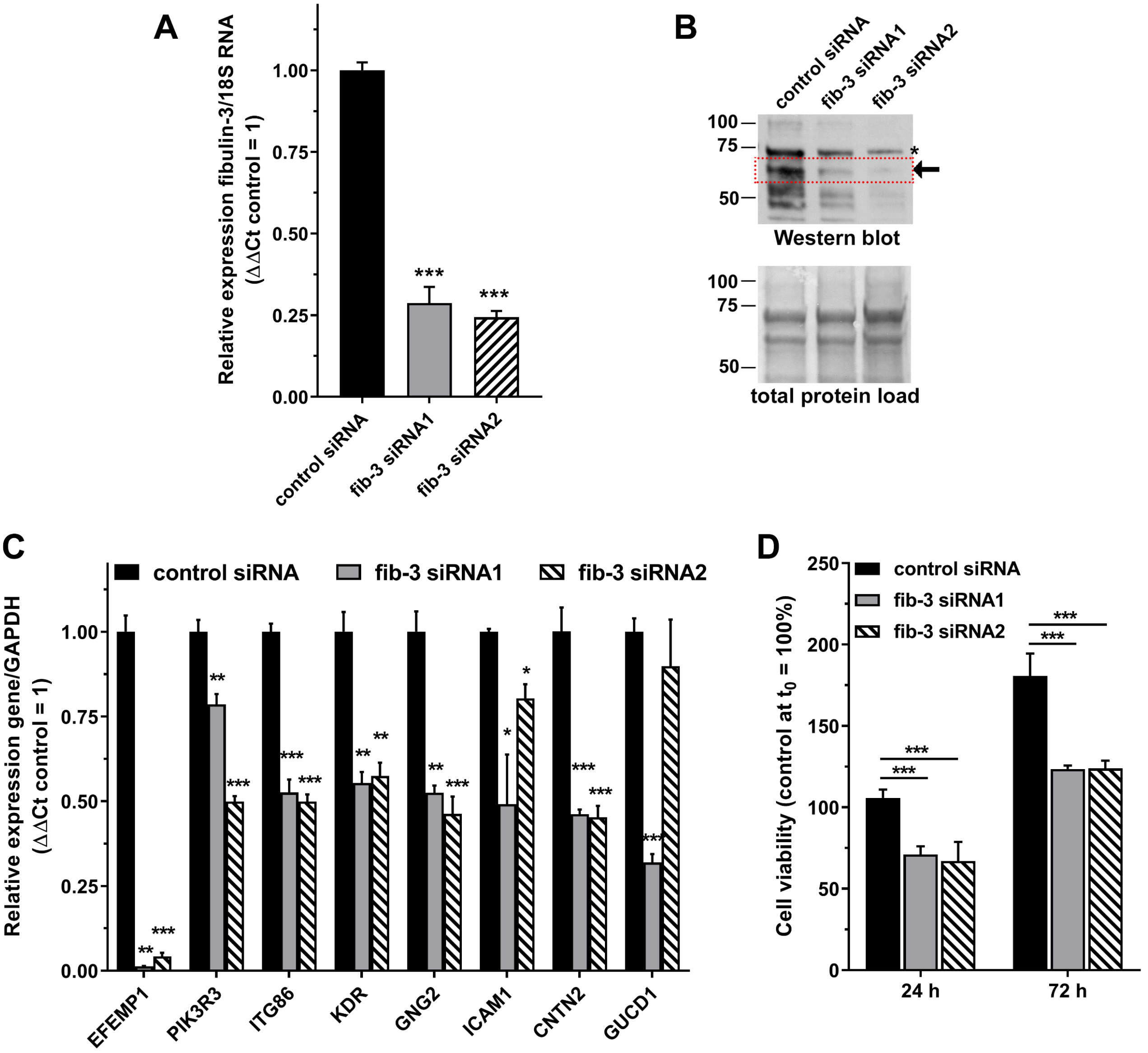


**Figure S1:** *Validation of fibulin-3 knockdown in MPM cells.* **A)** Confirmation of fibulin-3 knockdown in H2595 MPM cells by qRT-PCR using two independent RNAi sequences and a non-targeting negative control (Qiagen); *** p< 0.001 by one-way ANOVA. **B)** Confirmation of fibulin-3 knockdown by Western blotting; total protein load for culture medium containing secreted fibulin-3 was quantified by Bradford assay and confirmed by amido-black staining of the blot membrane before probing with antibody. Arrow: fibulin-3; asterisk: non-specific cross-reactivity. **C)** Knockdown of fibulin-3 with two independent RNAi sequences downregulates PI3K-dependent genes that are downstream of PI3K activation by fibulin-3 (shown in Figure 4) * p<0.05, ** p<0.01, *** p<0.001 by two-way ANOVA. **D)** Knockdown of fibulin-3 with two independent RNAi sequences abolish the enhancing effect of fibulin-3 on MPM cell proliferation; *** p<0.001 by two-way ANOVA.

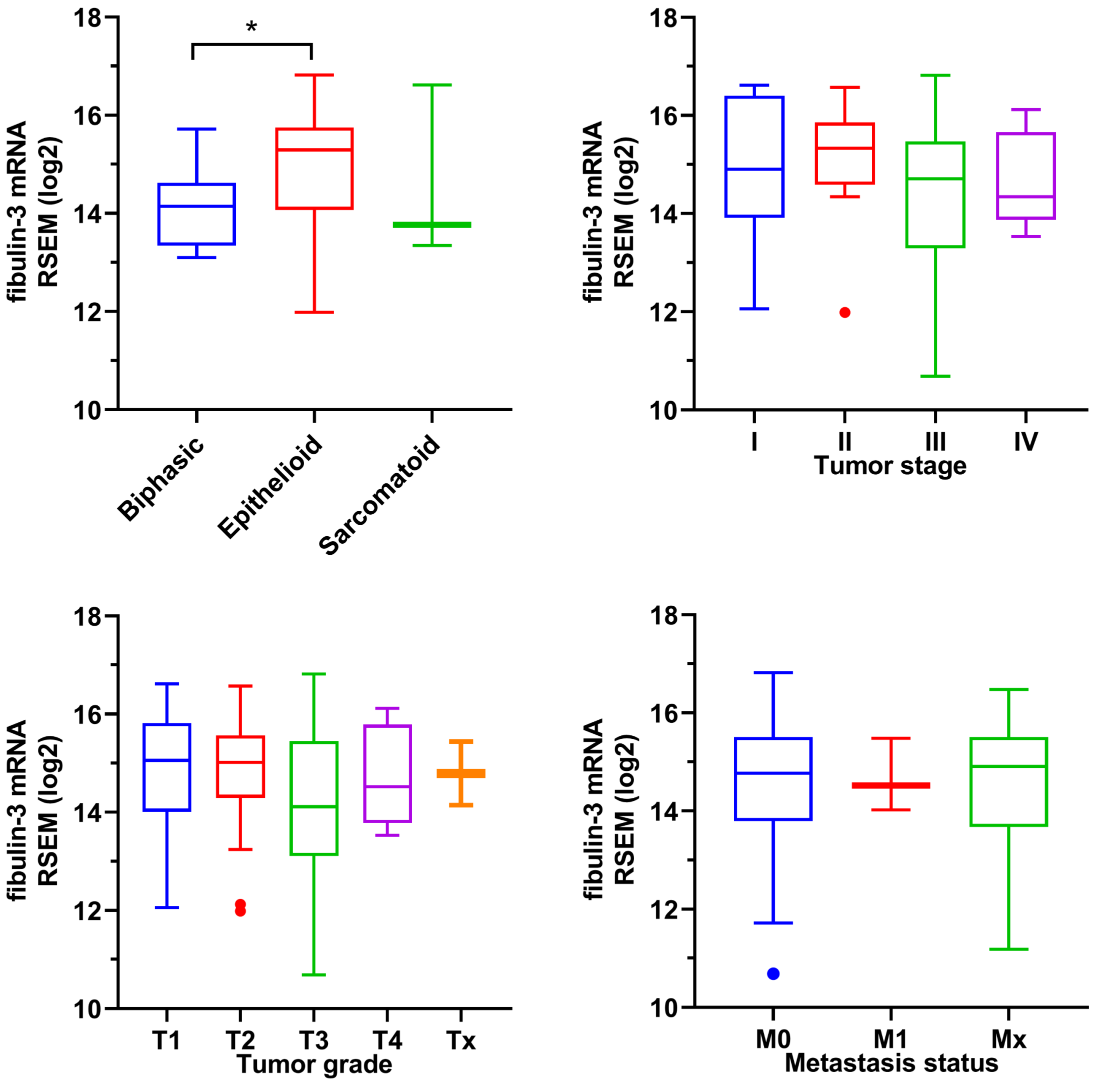


**Figure S2*:*** *Fibulin-3 expression does not correlate with diagnostic variables in MPM patients*. Fibulin-3 mRNA expression and clinical parameters were queried from the TCGA mesothelioma study (N=87) described by Hmeljak et al., *Cancer Discovery* (2018) 12:1548-1565. Fibulin-3 expression was higher in epithelioid than in biphasic or sarcoid mesotheliomas (* $p < 0.05$, 1-way ANOVA), but did not show significant differences among tumors of different grade, stage, or metastatic status. Data in the panels is represented by Tukey's box-plots showing median expression and interquartile distance; individual points indicate outliers.

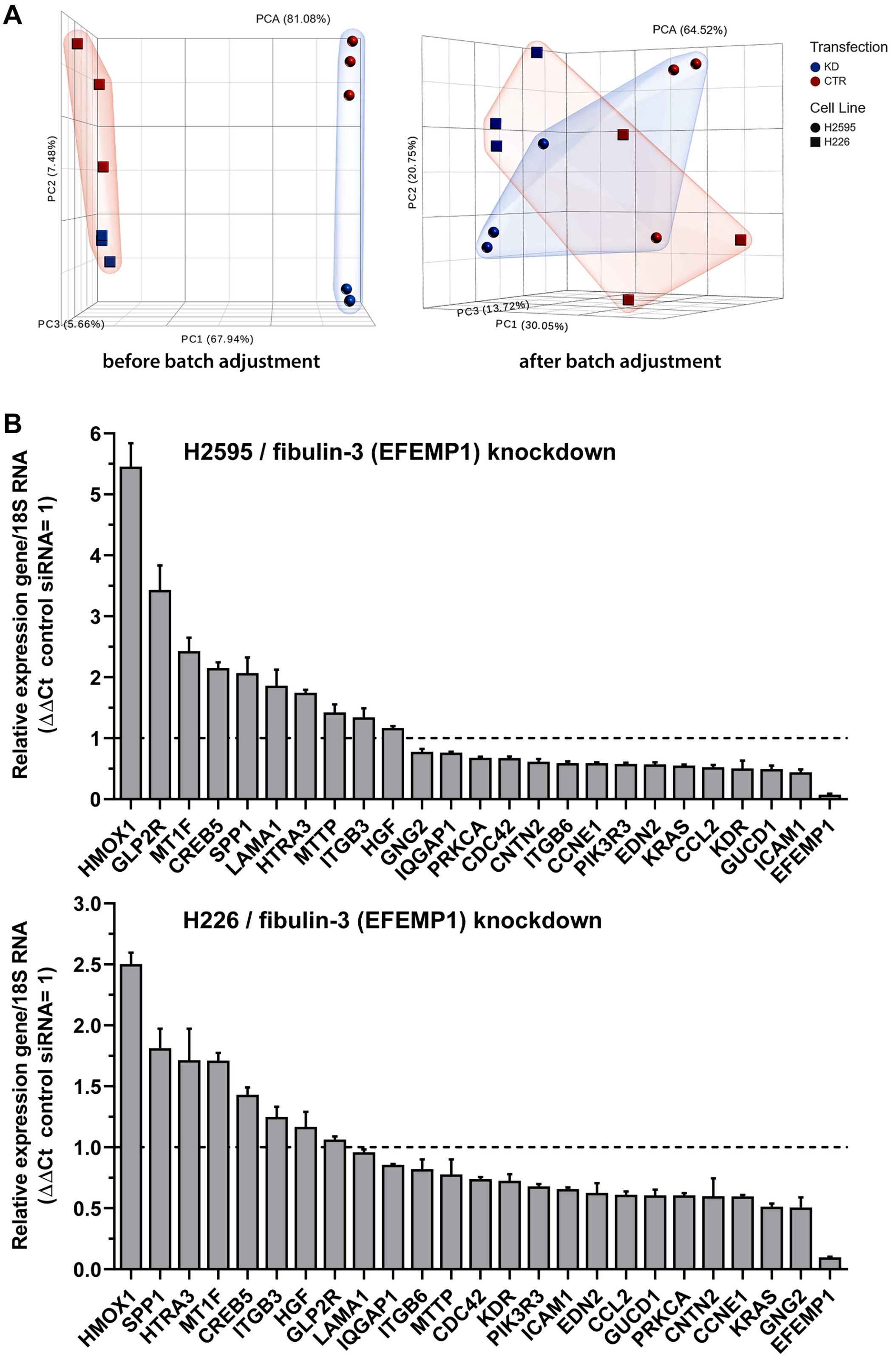


**Figure S3:** *Validation of RNAseq results after fibulin-3 knockdown.* **A**) Principal component analysis from the RNAseq experiment shown in Figure 3, before and after removal of cell line-dependent effects (batch effect adjustment). Notice how the major contributor to total variance before adjustment is the

overall difference between the two cell lines. Adjustment for gene expression differences between cell lines allowed the identification of common genes altered by fibulin-3 knockdown in both cell lines. **B**) qRT-PCR validation of a subset of 25 genes identified as up- or down-regulated by RNAseq after fibulin-3 knockdown. The graphs show the relative up- or down-regulation of those genes in fibulin-3-deficient cells (control ΔΔCt values were scaled equal to 1 for each gene). Notice the similar downregulation in both cell lines of multiple genes associated with PI3K signaling and cell adhesion, such as *GNG2*, *IQGAP1*, *CDC42*, *CNTN2*, *ITGB6*, *PIK3R3*, *EDN2*, *KRAS*, *KDR*, and *ICAM1*. *EFEMP1* = fibulin-3.
