# Supplementary material for: The extracellular matrix protein fibulin-3/EFEMP1 promotes pleural mesothelioma growth by activation of PI3K/Akt signaling
Source: Front Oncol. 2022 Oct 11;12:1014749. doi: 10.3389/fonc.2022.1014749 (PMC9593058; doi:10.3389/fonc.2022.1014749)
Supplement: Supplementary file 2 [file DataSheet_2.pdf]

Roshini et al.

Supplementary Table I SNP characterization of cells used in the study

Cell Line	Origin	Repository source	Tumor type	STR							
				Amelogenin	CSF1PO	D3S1358	D5S818	D7S820	D8S1179	D13S317	D16S539
LP-9	Female 26Y	Coriell Institute	Normal	X,X	10,11	15,17	11,12	11,12	9,10	8,12	10,11
H226	Male, adult	ATCC	Epithelioid	X,Y	10,11	16	11,12	8,10	14,15	13,14	9,12
MSTO-211H	Male, 62Y	ATCC	Biphasic	X,Y	11,11	15,15	12,12	8,12	13,13	11,14	13,13
H2595	Male, adult	Harvey Pass	Epithelioid	X,Y	11,11	18,18	13,13	7,9	10,14	11,12	11,13
H2596	Male, adult	Harvey Pass	Sarcomatoid	X,Y	11,11	16,16	12,12	9,11	13,16	13,13	12,12
H2452	Male, adult	Harvey Pass	Biphasic	X,Y	11,12	17,17	11,12	9,11	10,11	12,12	11,13
H2373	Female	Harvey Pass	Sarcomatoid	X,X	11,12	16,18	11,13	8,11	12,15	9,12	12,12
H2461	Female	Harvey Pass	Epithelioid	X,X	12,12	16,16	12,12	9,10	11,12	12,12	9,11
HP1	Male, adult	Harvey Pass	Biphasic	X,Y	10,10	17,17	11,13	8,9	14,16	9,12	12,12
HP3	Male, adult	Harvey Pass	Epithelioid	X,Y	11,12	17,17	11,12	11,13	10,11	12,12	11,13

Cell Line	Origin	Repository source	Tumor type	STR							
				D18S51	D21S11	FGA	Penta D	Penta E	TH01	TPOX	vWA
LP-9	Female 26Y	Coriell Institute	Normal	14,15	29,32.2	24,24	11,13	12,13	6,7	8,8	16,18
H226	Male, adult	ATCC	Epithelioid	16	29,32.2	20,23	9,10	12,15	8,9.3	8,11	17
MSTO-211H	Male, 62Y	ATCC	Biphasic	16,18	28,31	21,21	11,12	7,13	8,9.3	11,11	16,118
H2595	Male, adult	Harvey Pass	Epithelioid	12,24	29,31.2	23,25	13,14	11,13	7,7	11,11	15,16
H2596	Male, adult	Harvey Pass	Sarcomatoid	14,14	29,33.2	20,20	12,13	12,12	6,6	8,8	15,16
H2452	Male, adult	Harvey Pass	Biphasic	15,15	28,32.2	23,23	9,9	12,15	6,9.3	8,11	17,18
H2373	Female	Harvey Pass	Sarcomatoid	17,17	30,30	19,22	12,12	5,11	10,10	8,8	14,18
H2461	Female	Harvey Pass	Epithelioid	15,15	30,30	20,20	12,12	10,11	6,9	8,10	17,17
HP1	Male, adult	Harvey Pass	Biphasic	13,17	28,30	21,21	10,12	7,7	7,8	8,11	14,15
HP3	Male, adult	Harvey Pass	Epithelioid	15,15	28,32.2	23,23	9,9	12,15	6,9.3	8,11	17,18

Supplementary Table II: Sequences of fibulin-3 siRNAs and primers for qRT-PCR

**Gene**	**Sequence**	**Species**
Fibulin-3 siRNA1	5'-CGCAAUGCACUGACGGAUATT	human
Fibulin-3 siRNA2	5'-CAACGUGUGCCAAGACAUATT	human
Fibulin-3 forward	5'-TTTTGCTGTGCTGTGCAAGG	human
Fibulin-3 reverse	5'-CAGTGCATTGCGTGTACGTG	human
EDN2 forward	5'-AGGGACATTTCCACAGTCAAG	human
EDN2 reverse	5'-TCCAGCTCACGACACTATCT	human
CNTN2 forward	5'-GCAGTCACCGGCTATAAGATG	human
CNTN2 reverse	5'-GTCTTCAGGCACTGGGATTT	human
HMOX1 forward	5'-TCTTGGCTGGCTTCCTTAC	human
HMOX1 reverse	5'-CATAGGCTCCTTCCTCCTTTC	human
SPP1 forward	5'-GAAGTTTCGCAGACCTGACAT	human
SPP1 reverse	5'-GTATGCACCATTCAACTCCTCG	human
HTRA3 forward	5'-TGGCATCAACACGCTCAA	human
HTRA3 reverse	5'-GTCTTGGAACTCTGTGAGGAAC	human
MT1F forward	5'-GACAACCTTTCTCCCAGATGTA	human
MT1F reverse	5'-GGAATGTAGCAAATGGGTCAAG	human
GLP2R forward	5'-GTCCCAGGAAGTTGACCTATAAC	human
GLP2R reverse	5'-GCCCTCCTGCTTTCAGTATT	human
MTTP forward	5'-ACAAGCTCACGTACTCCACTG	human
MTTP reverse	5'-TCCTCCATAGTAAGGCCACATC	human
CREB5 forward	5'-ACACACAGCCTTTCCCATAC	human
CREB5 reverse	5'-AAGAGGAGGAGAGGTGAAGAG	human
LAMA1 forward	5'-TTAGCCACCGGGAACCTAAAG	human

LAMA1 reverse	5'-GCCATAGCAGATACACATGCCT	human
ITGB3 forward	5'- AGAAGCCAGCTTTCCTCATC	human
ITGB3 reverse	5'-AGCAGTTCCTACCATAGCTG	human
HGF forward	5'-GCTATCGGGGTAAAGACCTACA	human
HGF reverse	5'-CGTAGCGTACCTCTGGATTGC	human
GNG2 forward	5'-CCTTTCTAGTCCACGGCATTT	human
GNG2 reverse	5'-GGATCGGCTTGCATTCTCATA	human
IQGAP1 forward	5'-GTGGAGAGGATACAAGCAGAAG	human
IQGAP1 reverse	5'-TGCCAGGGACTGAATCTTTAC	human
PRKCA forward	5'-AGTTACGTCTGGCTCTAGGT	human
PRKCA reverse	5'-CACAGATGGAGGGTATGGAAAG	human
CDC42 forward	5'-GATTACGACCGCTGAGTTATCC	human
CDC42 reverse	5'-GTTATCTCAGGCACCCACTTT	human
ITGB6 forward	5'-CTCAACACAATAAAGGAGCTGGG	human
ITGB6 reverse	5'-AAAGGGGATACAGGTTTTTCCAC	human
CCNE1 forward	5'-GTACTGAGCTGGGCAAATAGAG	human
CCNE1 reverse	5'-GAAGAGGGTGTTGCTCAAGAA	human
PIK3R3 forward	5'-CTTTGCGGAAGGGAGGCAATA	human
PIK3R3 reverse	5'-ACCACGGAATTAAATGTCAGAGG	human
KRAS forward	5'-TGCTCCATGCAGACTGTTAG	human
KRAS reverse	5'-CTCTGGGAATACTGGCACTTAG	human
CCL2 forward	5'-TCATAGCAGCCACCTTCATTC	human
CCL2 reverse	5'-CTCTGCACTGAGATCTTCCTATTG	human
KDR forward	5'-GGCCCAATAATCAGAGTGGCA	human
KDR reverse	5'-CCAGTGTCATTTCCGATCACTTT	human
GUCD1 forward	5'-GACGACAGTGAGTTTGAGAGAG	human

GUCD1 reverse	5’-AAAGTGGTGCATCAGGTAGG	human
ICAM1 forward	5’-TTGGGCATAGAGACCCCGTT	human
ICAM1 reverse	5’-GCACATTGCTCAGTTCATACACC	human
TNC forward	5’-TTCAGCAGAATTGGGGATTT	human
TNC reverse	5’-ACCTAGGTCTCTCGCCCATC	human
MLCK forward	5’-CTGGGAAGCTCCTGAAGTTG	human
MLCK reverse	5’-GCGGGAAGGATTCTTCAAAA	human
MMP9 forward	5’-TCATCCAGTTTGGTGTCGCG	human / rat / mouse
MMP9 reverse	5’-GACCACAACTCGTCGTCGTC	human / rat / mouse
MMP13 forward	5’-GAGGTGACTGGCAAACTTGA	human / rat / mouse
MMP13 reverse	5’-ATATCAGGGGTGTAATTCAC	human / rat / mouse
ADAM17 forward	5’-TCATTGACCAGCTGAGCATC	human
ADAM17 reverse	5’-CAAGAATGCTGAAAGGAATCC	human
RELA forward	5’-ACTGCCGAGCTCAAGATCTG	universal
RELA reverse	5’-TCCCGTGAAATACACCTCAA	universal
18S RNA forward	5’-AACTTTCGATGGTAGTCGCCG	universal
18S RNA reverse	5’-CCTTGGATGTGGTAGCCGTTT	universal

Roshini et al.

Supplementary Table III: Antibodies used for Western blot blotting and immunohistochemistry

Target	Vendor	Catalog number	Host species	Target species	Application
Fibulin-3	Santa Cruz Biotechnology	SC-33722 (mAb3-5)	Mouse	Human, mouse	Western blot / IHC
Fibulin-3	Absolute Antibody	mAb428.2 (custom)	mouse-human IgG1 chimera	Human	Western blot / IHC
RelA/p65	Cell Signaling Technology	8242	Rabbit	Human, mouse, rat	Western blot
phospho-RelA/p65	Cell Signaling Technology	3037	Rabbit	Human, mouse, rat	Western blot
total PI3K	Cell Signaling Technology	4292	Rabbit	Human, mouse,rat	Western blot, Fig. 4A
total PI3K	Cell Signaling Technology	4257	Rabbit	Human, mouse,rat	Western blot, Fig. 4B
phospho-PI3K (Tyr458/Tyr199)	Cell Signaling Technology	4228	Rabbit	Mouse, tested against human	Western blot
total AKT	Cell Signaling Technology	9272	Rabbit	Human, mouse,rat, monkey	Western blot
phospho-AKT(Ser473)	Cell Signaling Technology	4058	Rabbit	Human, mouse,rat	Western blot
total MAPK p38	Cell Signaling Technology	8690	Rabbit	Human, mouse,rat, monkey	Western blot
phospho-p38 MAPK (Thr180/Tyr182)	Cell Signaling Technology	4511	Rabbit	Human, mouse,rat, monkey	Western blot
Ki67	Thermo Scientific	RM-9106	Rabbit	Human	IHC
IKK α	Cell Signaling Technology	2682	Rabbit	Human, mouse,rat, monkey	Western blot
IKK β	Cell Signaling Technology	2678	Rabbit	Human, mouse,rat, monkey	Western blot

phospho-IKK αβ	Cell Signaling Technology	2697	Rabbit	Human, mouse,rat, monkey	Western blot
IκB	Cell Signaling Technology	4812	Rabbit	Human, mouse,rat, monkey	Western blot
beta Actin	Cell Signaling Technology	4970	Rabbit	Human, mouse,rat, monkey	Western blot
alpha/beta tubulin	Cell Signaling Technology	2148	Rabbit	Human, mouse, rat, monkey	Western blot

<u>*Supplementary Table IV:*</u> Abbreviated names of TCGA studies / cancer types

**TCGA study abbreviation**	**Study name (cancer type)**	**Number of cases**
ACC	Adrenocortical carcinoma	92
BLCA	Bladder Urothelial Carcinoma	412
BRCA	Breast invasive carcinoma	1098
CESC	Cervical squamous cell carcinoma and endocervical adenocarcinoma	307
CHOL	Cholangiocarcinoma	51
COAD	Colon adenocarcinoma	460
COADREAD	Colorectal adenocarcinoma	631
DLBC	Lymphoid Neoplasm Diffuse Large B-cell Lymphoma	58
ESCA	Esophageal carcinoma	185
GBM	Glioblastoma multiforme	613
GBMLGG	High and low-grade gliomas	1129
HNSC	Head and Neck squamous cell carcinoma	528
KICH	Kidney Chromophobe	113
KIPAN	Pan-kidney cohort (KICH+KIRC+KIRP)	973
KIRC	Kidney renal clear cell carcinoma	537
KIRP	Kidney renal papillary cell carcinoma	323
LAML	Acute Myeloid Leukemia	200
LGG	Brain Lower Grade Glioma	516
LIHC	Liver hepatocellular carcinoma	377
LUAD	Lung adenocarcinoma	585
LUSC	Lung squamous cell carcinoma	504
**MESO**	**Mesothelioma**	**87**
OV	Ovarian serous cystadenocarcinoma	602
PAAD	Pancreatic adenocarcinoma	185
PCPG	Pheochromocytoma and Paraganglioma	179
PRAD	Prostate adenocarcinoma	499
READ	Rectum adenocarcinoma	171
SARC	Sarcoma	261
SKCM	Skin Cutaneous Melanoma	470
STAD	Stomach adenocarcinoma	443
STES	Stomach and Esophageal carcinoma	628
TGCT	Testicular Germ Cell Tumors	150
THCA	Thyroid carcinoma	503
THYM	Thymoma	124
UCEC	Uterine Corpus Endometrial Carcinoma	560
UCS	Uterine Carcinosarcoma	57
UVM	Uveal Melanoma	80
